# Genetic and antigenic divergence in the influenza A(H3N2) virus circulating between 2016 and 2017 in Thailand

**DOI:** 10.1371/journal.pone.0189511

**Published:** 2017-12-18

**Authors:** Nungruthai Suntronwong, Sirapa Klinfueng, Preeyaporn Vichiwattana, Sumeth Korkong, Thanunrat Thongmee, Sompong Vongpunsawad, Yong Poovorawan

**Affiliations:** Center of Excellence in Clinical Virology, Department of Pediatrics, Faculty of Medicine, Chulalongkorn University, Bangkok, Thailand; St. Jude Children’s Research Hospital, UNITED STATES

## Abstract

Influenza virus evolves rapidly due to the accumulated genetic variations on the viral sequence. Unlike in North America and Europe, influenza season in the tropical Southeast Asia spans both the rainy and cool seasons. Thus, influenza epidemiology and viral evolution sometimes differ from other regions, which affect the ever-changing efficacy of the vaccine. To monitor the current circulating influenza viruses in this region, we determined the predominant influenza virus strains circulating in Thailand between January 2016 and June 2017 by screening 7,228 samples from patients with influenza-like illness. During this time, influenza A(H3N2) virus was the predominant influenza virus detected. We then phylogenetically compared the hemagglutinin (HA) gene from a subset of these A(H3N2) strains (n = 62) to the reference sequences and evaluated amino acid changes in the dominant antigenic epitopes on the HA protein structure. The divergence of the circulating A(H3N2) from the A/Hong Kong/4801/2014 vaccine strain formed five genetic groups (designated I to V) within the 3C.2a clade. Our results suggest a marked drift of the current circulating A(H3N2) strains in Thailand, which collectively contributed to the declining predicted vaccine effectiveness (VE) from 74% in 2016 down to 48% in 2017.

## Introduction

Influenza A virus is an important respiratory pathogen responsible for the annual influenza outbreak and considerable socio-economic burden on the public healthcare system [1]. The multivalent influenza virus vaccine administered annually can reduce the risk of morbidity and mortality, but it is dependent on how well the chosen strains included in the vaccine match the strains in circulation [2]. The commonly circulating seasonal influenza A subtypes are A(H1N1)pdm09 and A(H3N2), of which the latter is reportedly associated with a high rate of hospitalization and mortality in the United States in the 2016–2017 flu season [3].

The hemagglutinin (HA) surface glycoprotein of A(H3N2) possesses defined antigenic and receptor-binding sites [4,5]. The HA diversity resulting from accumulated mutations facilitates viral escape from the host immune response [6,7]. The HA protein is proteolytically processed into two subunits (HA1 and HA2) [8]. The globular HA1 domain contains five antigenic sites (A through E) [9–11], while the HA2 stem domain mediates fusion and viral uncoating [12,13]. Sequence drifts on the HA from accumulated mutations are observed more frequently in the A(H3N2) than A(H1N1), which often lead to the gradual reduction of the vaccine effectiveness (VE) over time [14–16]. As a result, influenza virus strains most suitable for vaccine production are carefully evaluated each year [17,18].

Timely analysis of the genetic variations on the HA1 sequence of the circulating influenza virus strains is crucial for the prediction of VE. We therefore determined whether A(H3N2) was regionally predominant in the current influenza season and compared the genetic composition of the circulating A(H3N2) to the current vaccine strain A/Hong Kong/4801/2014.

## Materials and methods

### Ethical approval

Respiratory samples from patients with influenza-like illness were analyzed in the Center of Excellence in Clinical Virology at King Chulalongkorn Memorial Hospital as part of the routine influenza surveillance program. This study was approved by the Institutional Review Board (IRB) of the Faculty of Medicine at Chulalongkorn University (IRB No. 377/57). The IRB waived the need for consent because the samples were de-identified and anonymous.

### Samples and HA gene amplification

A total of 7,228 samples obtained between January 2016 and June 2017 in Bangkok and Khon Kaen province were collected from patients with fever >38°C and respiratory symptoms such as sore throat, nasal congestion, cough and runny nose. These individuals sought medical care at King Chulalongkorn Memorial Hospital, Bangpakok 9 International Hospital, and Chum Phae Hospital. Samples were subjected to viral RNA extraction (GeneAll Biotechnology, Seoul, Korea) according to the manufacturer’s instructions. We used a previously described real-time reverse-transcription polymerase chain reaction (RT-PCR) to identify influenza virus A(H1N1pdm09), A(H3N2), and influenza B virus [19]. Influenza B virus-positive samples were subjected to cDNA synthesis using ImProm-II reverse transcription system (Promega, Madison, WI) and primer FluB (5’-AGCAGAAGCA-3’) [20], followed by lineage determination using multiplex PCR and melting curve analysis [21,22]. Among A(H3N2)-positive samples, 62 strains (approximately 4 strains per month) were randomly selected for cDNA synthesis using primer Uni12 (5’-AGCAAAAGCAGG-3’) [23] and the entire HA gene amplified using published primer sets [15]. Briefly, the reaction mixture consisted of 5 μl PRIME MasterMix (5Prime, Hamburg, Germany), 0.25 mM of MgCl_2_, 0.5 μM each of forward and reverse primers, 2 μl of cDNA template, and nuclease-free water to a final volume of 25 μl. Amplification in a thermal cycler was performed under the following conditions: initial denaturation for 3 minutes at 94°C, followed by 40 cycles of 30 seconds at 94°C for denaturation, primer annealing for 30 seconds at 55°C, 90 seconds at 72°C for extension, and 7 minutes of final extension at 72°C. Amplicons were agarose gel-purified using Expin Combo GP kit (GeneAll Biotechnology, Seoul, Korea) and the HA gene sequenced. A(H3N2) nucleotide sequences were assembled using the SeqMan Pro software (DNASTAR, Madison, WI) and deposited in the GenBank database under the accession numbers (MF673231-MF673292) (S1 Table).

### Phylogenetic analysis

A total of 91 A(H3N2) HA nucleotide sequences, 62 obtained from this study and an additional 29 sequences identified in Thailand publicly available from the NCBI (http://www.ncbi.nlm.nih.gov) and GISAID (http://platform.gisaid.org) databases, were aligned using ClustalW and translated into amino acid residues using BioEdit Software version 7.0.9.1 (S2 Table). Phylogenetic analysis was performed using the maximum likelihood method and HKY+G model implemented in MEGA 6 [24]. Bootstrapping was done in 1,000 replicates and values >70% were shown. Potential N-linked glycosylation sites on the HA was determined using the NetNGlyc 1.0 server with the threshold value of >0.5 for the mean potential score [25]. The selective pressure or the proportion between non-synonymous to synonymous substitutions (d*N*/d*S*, defined as ω) observed on all HA sequences were analyzed using the single likelihood ancestor counting (SLAC), fixed effects likelihood (FEL), and mixed effects model of evolution (MEME) algorithms implemented in the HYPHY software [26]. Positively selected codon was considered significant at *P*-value of 0.1. Amino acid residue numbering was based on the HA1 of the A(H3N2) vaccine strain A/Hong Kong/4801/2014 unless otherwise indicated. Residues different from those of the A/Hong Kong/4801/2014 were placed on a three-dimensional HA protein structure (A/Aichi/2/1968; Protein Data Bank accession number 1HGE) using VMD 1.9.2 [27].

### Estimates of vaccine effectiveness

The predicted vaccine effectiveness (VE) was estimated using the *P*_epitope_ model, which characterizes the antigenic distance between the A(H3N2) vaccine and circulating strains. Antigenic distance defined by the *P*_epitope_ was calculated from the fraction of substituted amino acid residues in the dominant HA epitope [28]. For A(H3N2), the association between the VE and the *P*_epitope_ is given by VE = -2.47 × *P*_epitope_ + 0.47 in which VE is 47% when *P*_epitope_ = 0.

### Statistical analysis

Statistical analyses were performed using the Statistical Package for Social Sciences version 22.0 (SPSS Inc., Chicago, USA). The one-way ANOVA test was used to analyze VE divergence, and *p* < 0.05 was considered statistically significant.

## Results

**Fig 1:**
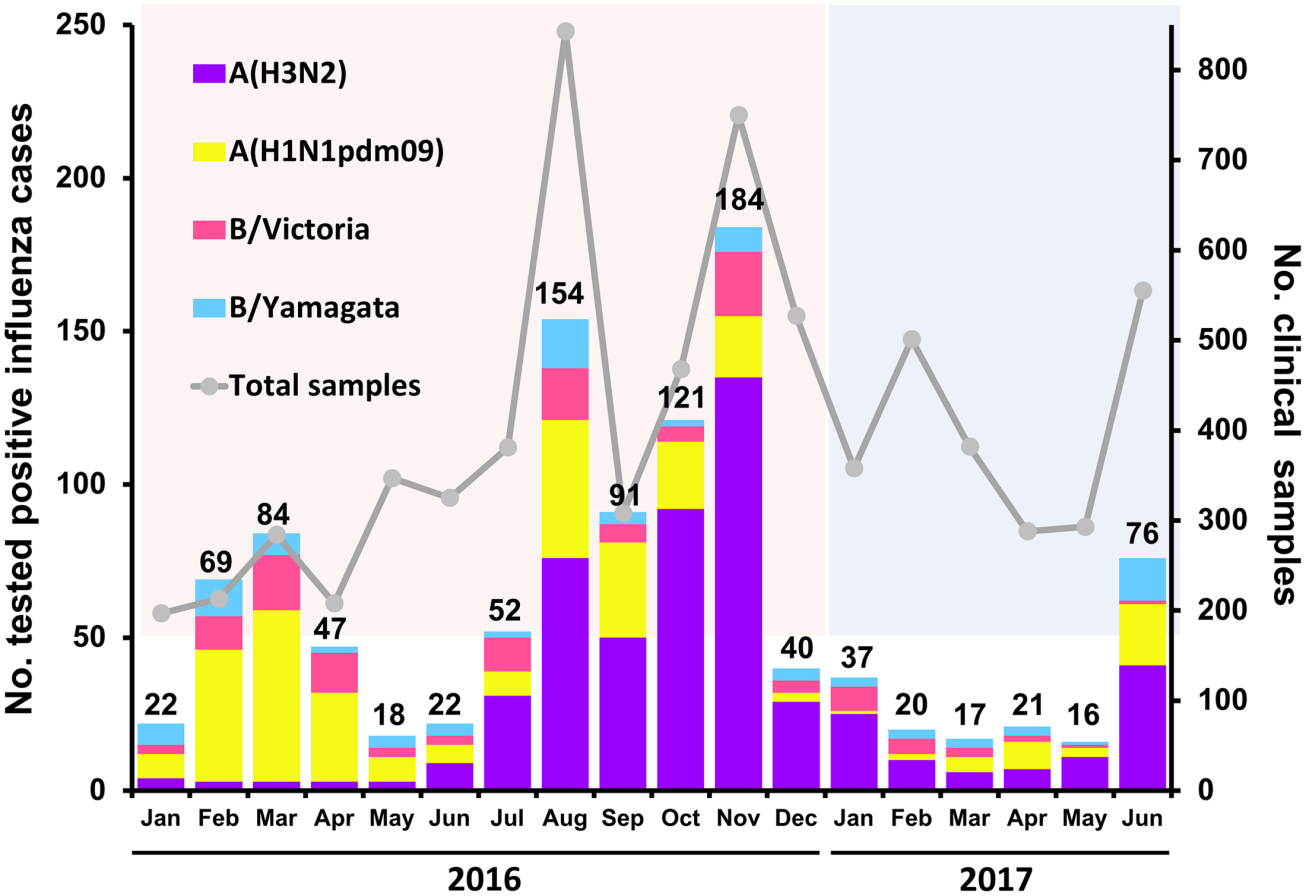
Distribution of influenza A(H3N2) virus between January 2016 and June 2017 (n = 7,228). Total number of clinical samples evaluated (Y-axis on the right) and different influenza virus-positive samples are shown (Y-axis on the left). Numbers above the bar graphs denote influenza virus-positive samples identified each month.

In all, 15.1% of the samples (1,091/7,228) tested positive for influenza viruses, of which 78.6% (857/1,091) were influenza A virus and 21.4% (234/1,091) were influenza B virus (Fig 1). As expected, an increase in the number of influenza virus-positive samples occurred in the rainier months (August to November). Among influenza A virus-positive samples, 62.8% (538/857) were A(H3N2) and 37.2% (319/857) were A(H1N1pdm09). Influenza B virus lineages were found to be 57.7% (135/234) B/Victoria and 42.3% (99/234) B/Yamagata. Thus, A(H3N2) represented the majority of the influenza virus during this study period.

### Analysis of the HA gene sequence

**Fig 2:**
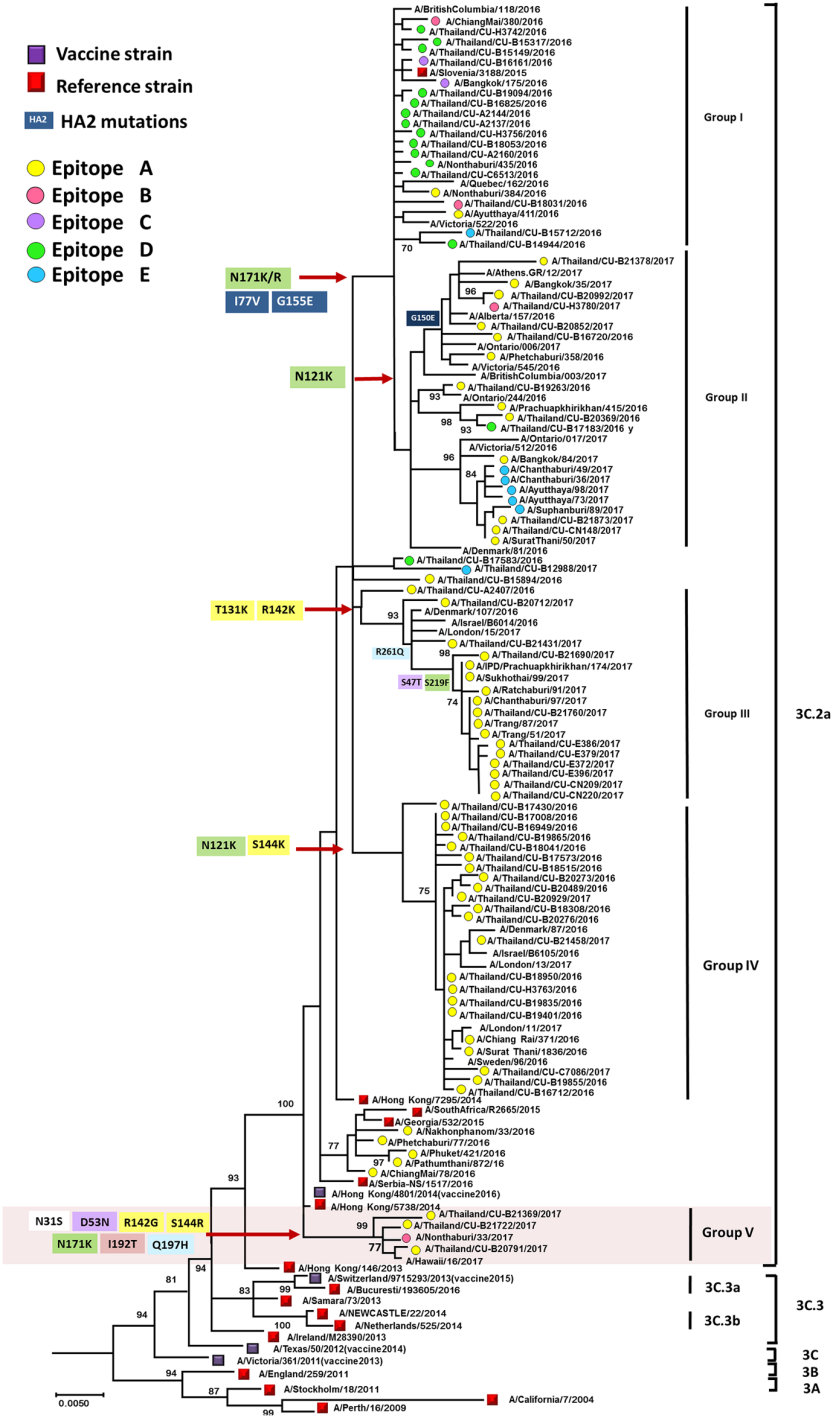
Phylogenetic analysis of the nucleotide sequences of the HA coding region of A(H3N2). Sequences of randomly selected samples from this study (n = 62) (designated A/Thailand/CU and denoted in colored dots) and those identified in Thailand from the databases during 2016–2017 (n = 29) were compared to the A(H3N2) vaccine and reference strains of known clades reported by the WHO and others (squares). The phylogenetic tree was constructed using the maximum likelihood method and HKY+G model with 1,000 bootstrap replicates implemented in MEGA (version 6). Branch values of >70% are indicated at the nodes. Dominant epitope (A-E) determined for each sequence are marked with different colored dots (A = yellow, B = pink, C = purple, D = green, and E = blue). The signature amino acid substitutions (colored) occurring on the antigenic epitopes are also shown. Scale bar represents approximately 0.5% nucleotide difference between close relatives. Residue numbers are specific for HA1 (color-coded by epitope) and HA2 (dark blue). The vaccine strain A/Hong Kong/4801/2014 belonged to 3C.2a clade. Shaded area (Group V) highlights strains of interest.

Ninety-one complete coding sequences of the HA gene showed between 98.5–99.6% nucleotide sequence identity (98.1–99.8% amino acid sequence identity) to the A/Hong Kong/4801/2014 vaccine strain for A(H3N2). Among these, the vast majority significantly diverged from the A/Hong Kong/4801/2014 (Fig 2). Most belonged to the 3C.2a clade, which possessed distinct variations in amino acid patterns (designated group I to V). Their divergence was associated with sequential overlapping time of circulation. For example, group I strains (20/91) circulated in January to October 2016, while group II strains (20/91) circulated in November 2016 to March 2017. Group III strains (16/91) circulated between January and June 2017. Group IV strains (22/91) circulated between September 2016 and March 2017 and were most closely related to the A(H3N2) strains identified during 2016–2017 influenza season from several countries in the northern hemisphere (A/Denmark/107/2016, A/Israel/B6014/2016, and A/London/15/2017) [29–31]. Interestingly, several novel A(H3N2) strains (4/91) circulating between January and April 2017 possessed sufficient residue variations in addition to what were previously described to warrant a separate cluster (group V).

### Residue variations on the HA affecting antigenic epitopes, receptor-binding site, and potential glycosylation

**Fig 3:**
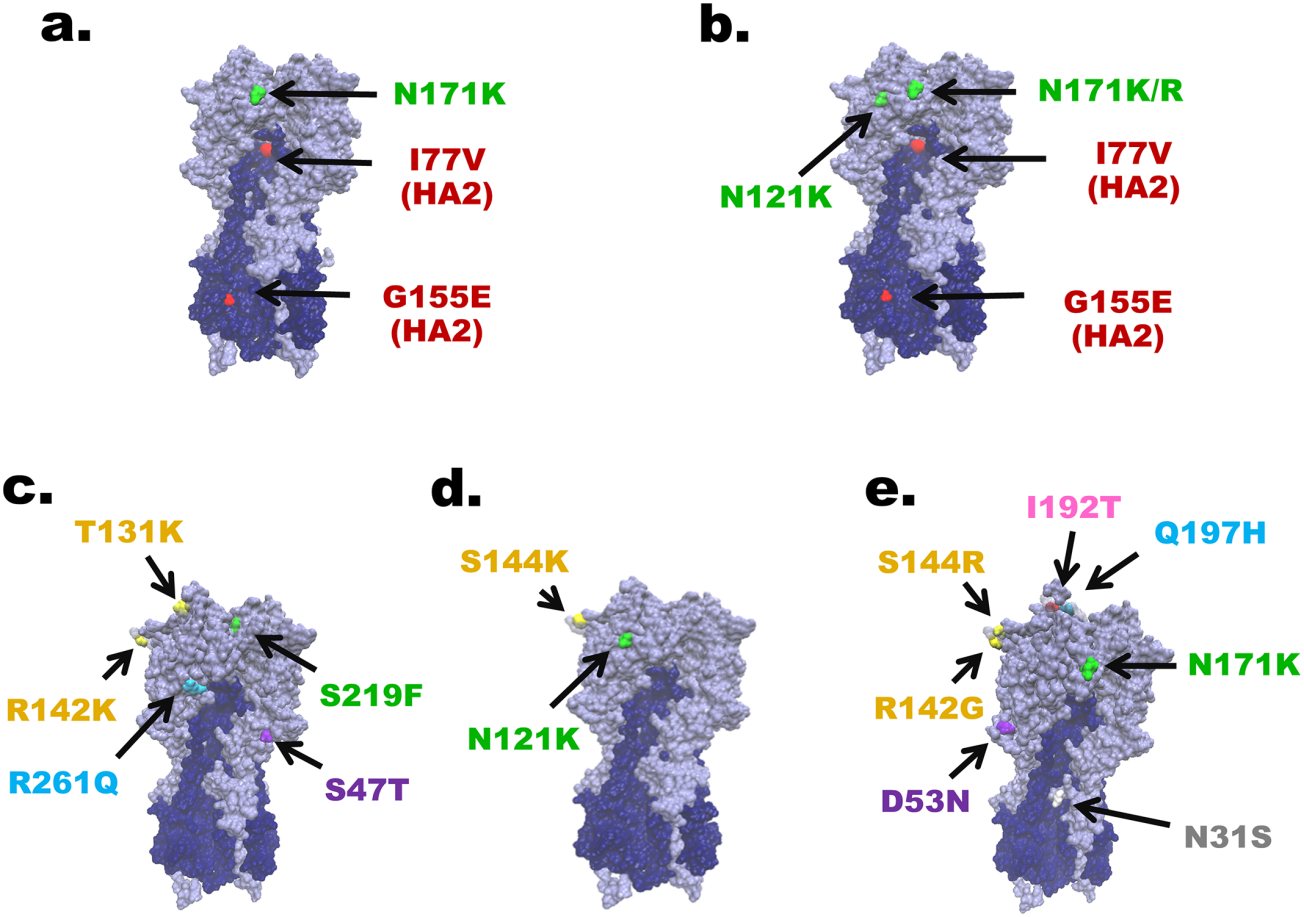
The defining residue substitutions placed on the HA protein structure of A(H3N2). Differences between A(H3N2) strains in this study and A/Hong Kong/4801/2014 were visualized on the homotrimeric HA structure of A/Aichi/2/1968 (Protein Data Bank accession number: 1HGE). (a) to (e) correspond to groups I to V, respectively. Residues on the five antigenic sites are color-coded: A (yellow), B (pink), C (purple), D (green) and E (blue). Mutation not located within the antigenic site (white) and mutations in HA2 (red) are also noted.

Comparison of the deduced amino acid sequences to that of A/Hong Kong/4801/2014 showed that group I differed from the vaccine strain by residues N171K and I77V+G155E(HA2), and group II by N121K+N171K/R and I77V+G155E(HA2). Some strains in group II also possessed additional residue variants K92R+H311Q (9/20), or G150E (HA2) (6/20) (S1 Fig). Group III strains were defined by residue pattern S47T+T131K+R142K+S219F+R261Q. Meanwhile, group IV strains differed from the vaccine strain by N121K+S144K. Most interestingly, the novel group V formed by strains identified in this study possessed a unique constellation of residue variations N31S+D53N+R142G+S144R+N171K+I192T+Q197H (Fig 3).

A(H3N2) strains circulating in Thailand collectively showed a total of 39 residue substitutions in the antigenic epitopes. Some of the strains possessed as many as seven amino acid changes (S1 Fig). Among 53 sequenced strains from 2016, those identified at the beginning of the year were epitope D dominant as characterized by N121K, N171K/R, and S219Y/F. Sequence shift to mostly epitope A were observed by the end of 2016 as characterized by N122D, T131K, T135K, R142K/G and S144K/R. Not surprisingly, A(H3N2) strains identified in the first-half of 2017 (n = 38) continued to show dominance in epitope A. Among the potential N-linked glycosylation sites on the HA (at residues 8, 22, 38, 45, 63,122, 126, 133, 158, 165, 246, 285 and 483 on the A/Hong Kong/4801/2014 vaccine strain) [15], substitutions at N122D (1/91) and S124N (2/91) found in some strains in this study eliminated the potential glycosylation site at residue 122 (S1 Fig). Additionally, there was a loss of potential glycosylation site at residue 126 due to N126S substitution (1/91), at residue 133 due to T135K (9/91), and at residue 158 due to N158K (3/91) and T160K (2/91) substitutions.

The receptor-binding sites of A(H3N2) are highly conserved at positions 98, 136, 153, 183, 190, 194, 195 and 228 on HA1 [13]. We observed T135K residue change adjacent to the receptor-binding region for all 3C.2a group II strains. Evolutionary selective pressure on the entire HA amino acid sequence analyzed using the d*N*/d*S* ratio showed an average of 0.305 (SLAC algorithm) among the A(H3N2) strains in this study, suggesting no positively selected site. Meanwhile, the FEL method revealed that residues 142, 171, 261 and 406 (HA numbering) were under positive selection pressure. Moreover, the MEME method indicated five positively selected sites (131, 144, 171, 261 and 416). Therefore, these data provided strong evidence of positively selected sites within epitope A (131, 142 and 144), D (171) and E (261) (S3 Table).

### Predicted vaccine effectiveness

**Fig 4:**
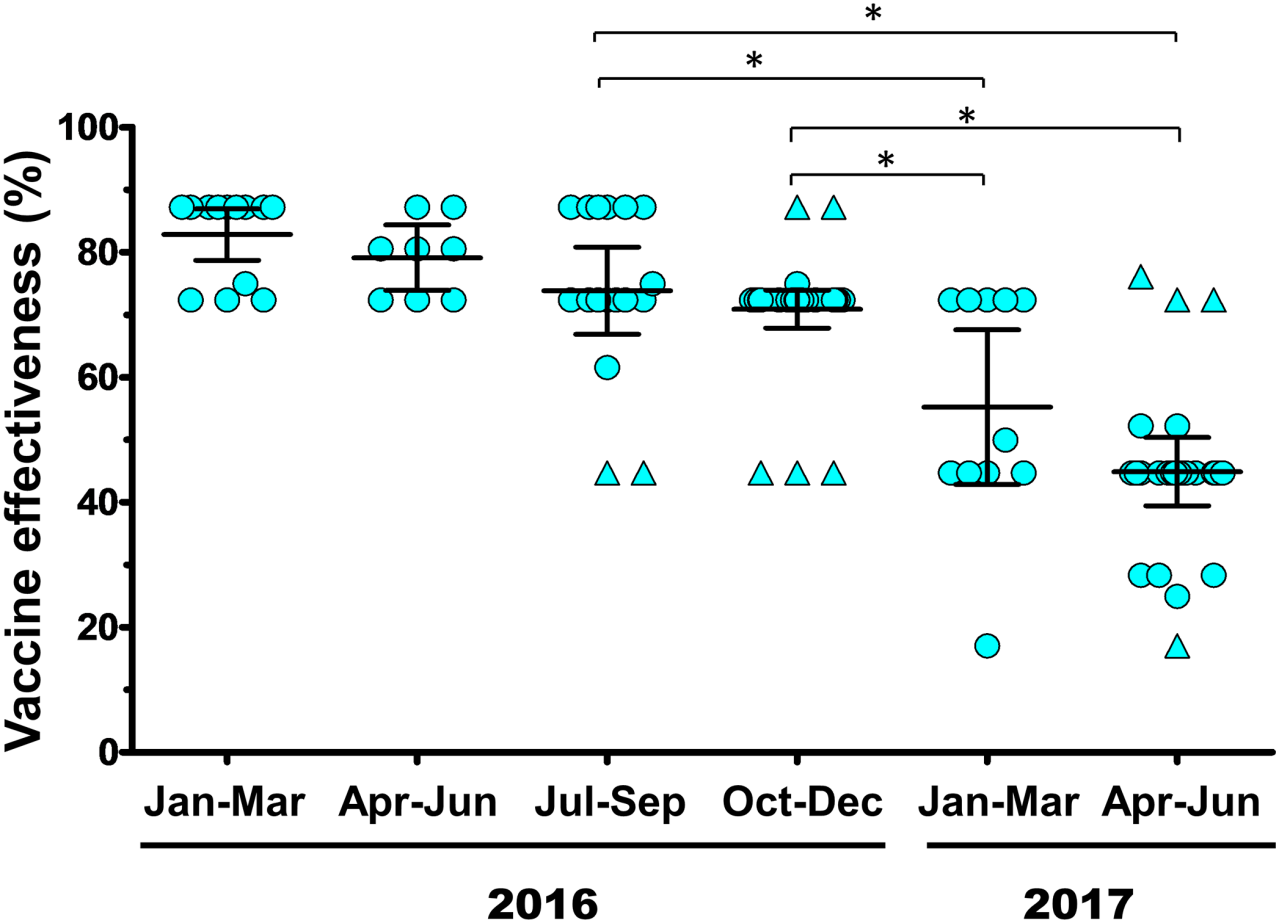
Calculated quarterly vaccine effectiveness between January 2016 and June 2017. Vaccine effectiveness (VE) was derived using the *P*_epitope_ model by comparing the A(H3N2) sequences identified in this study to that of the vaccine strain A/Hong Kong/4801/2014. Circles represent the predicted VE percentage for each strain analyzed, while triangles represent statistical outliers. For each quarter, the middle bar represents the mean value, while the upper and lower bars denote interquartile range. Asterisks indicate statistically significant differences (P<0.01).

The average *P*_epitope_ value was 0.049 (n = 53) for all of the A(H3N2) strains identified in 2016, suggesting a predicted VE against the virus of 74.17%. Meanwhile, the average *P*_epitope_ was 0.099 (n = 38) for strains identified in 2017, suggesting a predicted VE against the virus of 47.87% (S4 Table). Although the predicted VE between the strains identified in this study and the A/Hong Kong/4801/2014 vaccine strain ranged between 17.02 and 87.18%, there was an overall decline in predicted VE each quarter (Fig 4). These results suggest that the A(H3N2) strains circulating in Thailand drifted from the vaccine strain and effectively reduced the VE.

## Discussion

In this study, we aimed to characterize the circulating A(H3N2) in Thailand beginning in 2016 by determining clade designation and identifying mutations in the antigenic sites impacting the predicted VE. We categorized the strains identified in this study and those reported elsewhere between 2016 and 2017 using the HA sequences. Due to evolving genetic variations of A(H3N2) and the lagging nomenclature standard, we attempted to reconcile the most recent 3C.2a strains identified to date. Group I and II strains in this study collectively represented clade 3C.2a1 designated by the WHO [32]. Group III strains in this study comprised some newer Israel strains and the proposed 3C.2a1 cluster III from the U.K., while group IV described additional Israel strains and the proposed 3C.2a2 clade [30,31]. Additionally, newly emergent A(H3N2) strains identified in this study necessitated further subdivision into a separate group V according to a number of significant variance from the A/Hong Kong/4801/2014 vaccine strain and other previously described variants. Members of group V were characterized by seven amino acid substitutions at N31S, D53N, R142G, S144R, N171K, I192T and Q197H. These residue variants have a number of implications including alterations of the antigenic epitopes and immune escape. For example, residue 144 in antigenic site A is adjacent to residue S145 implicated in receptor-binding [33].

New antigenic variants emerge when at least one substitution occurred in the antigenic sites [11,34]. Several strains circulating in Thailand (5/91) belonging to group II and IV demonstrated F193S change located in epitope B, which is one of seven mutation sites representing a major antigenic transition cluster [5]. Both T135K and N126S in some A(H3N2) strains resulted in the loss of N-linked glycosylation, an important observation to consider since the gain or loss of N-linked glycosylation can affect influenza virus virulence and recognition by neutralizing antibody [35]. T135K located adjacent to the receptor-binding site in epitope A has also been identified in other studies and is implicated in decreasing vaccine effectiveness [36,37]. Taken together, these variations underscore the rapid evolution of A(H3N2) influenza virus in circulation.

Southeast Asian countries use the influenza vaccine formulated for the southern hemisphere, which for some years have different inclusion strains in the vaccine than those formulated for the northern hemisphere. A/Hong Kong/4801/2014 belonging to clade 3C.2a is a component in both northern and southern hemispheres since 2016. This vaccine strain was well-matched for the circulating A(H3N2) in Thailand that first season (predicted VE of approximately 80%) [38]. Since then, A(H3N2) strains circulating in Europe and Canada have genetically drifted away from the vaccine strain consistent with the observed antigenic drift and decreasing predicted VE we found in this study for each quarterly period beginning in 2016 [39–41]. As a result, A/Hong Kong/4801/2014 may not be as effective in eliciting immunity against future circulating A(H3N2) in the next influenza season despite the decision to include it in the 2017–2018 vaccine for northern hemisphere [42].

This study had several limitations. We were unable to ascertain the vaccination status of the individuals in which the samples were derived, which would have improved the evaluation of the predicted VE. The scope of this study did not allow us to determine additional evolutionary relationships among the strains identified in different countries, which would have required additional nucleotide sequences from around the world not yet deposited in the databases and a longer study time frame. The antigenic drift and predicted VE were estimated from the accumulated mutations on the antigenic epitopes and would benefit from additional antigenic characterization such as hemagglutination inhibition assay. Finally, immunity against the neuraminidase contributing to the antigenic drift was not assessed in this study. Nevertheless, any genetic surveillance of influenza viruses will continue to be an important component in influenza prevention and vaccine improvement.

## Supporting information

S1 Fig Amino acid substitutions found in 91 A(H3N2) Thai strains compared to A/Hong Kong/4801/2014 vaccine strain by major amino acid substitutions at the antigenic sites (A through E). Dominant epitopes (A through E) for each sequence are denoted in different colors (A = yellow, B = pink, C = purple, D = green, E = blue).
(TIF)

S1 Table Influenza A(H3N2) virus strains sequenced in this study. (DOCX)

S2 Table Accession numbers in GenBank and GISAID of HA influenza A(H3N2) and gene sequences used for phylogenetic tree analysis. (DOCX)

S3 Table Positive selected sites on the HA coding sequences of influenza A(H3N2) identified in Thailand between January 2016 and June 2017. (DOCX)

S4 Table Calculated vaccine efficacy using the *P*_epitope_ model and number of mutations in the dominant epitope of A(H3N2) strains in this study. (DOCX)
