# Supplementary figures and images for: Genetic and antigenic divergence in the influenza A(H3N2) virus circulating between 2016 and 2017 in Thailand
Source: PLoS One. 2017 Dec 18;12(12):e0189511. doi: 10.1371/journal.pone.0189511 (PMC5734729; doi:10.1371/journal.pone.0189511)

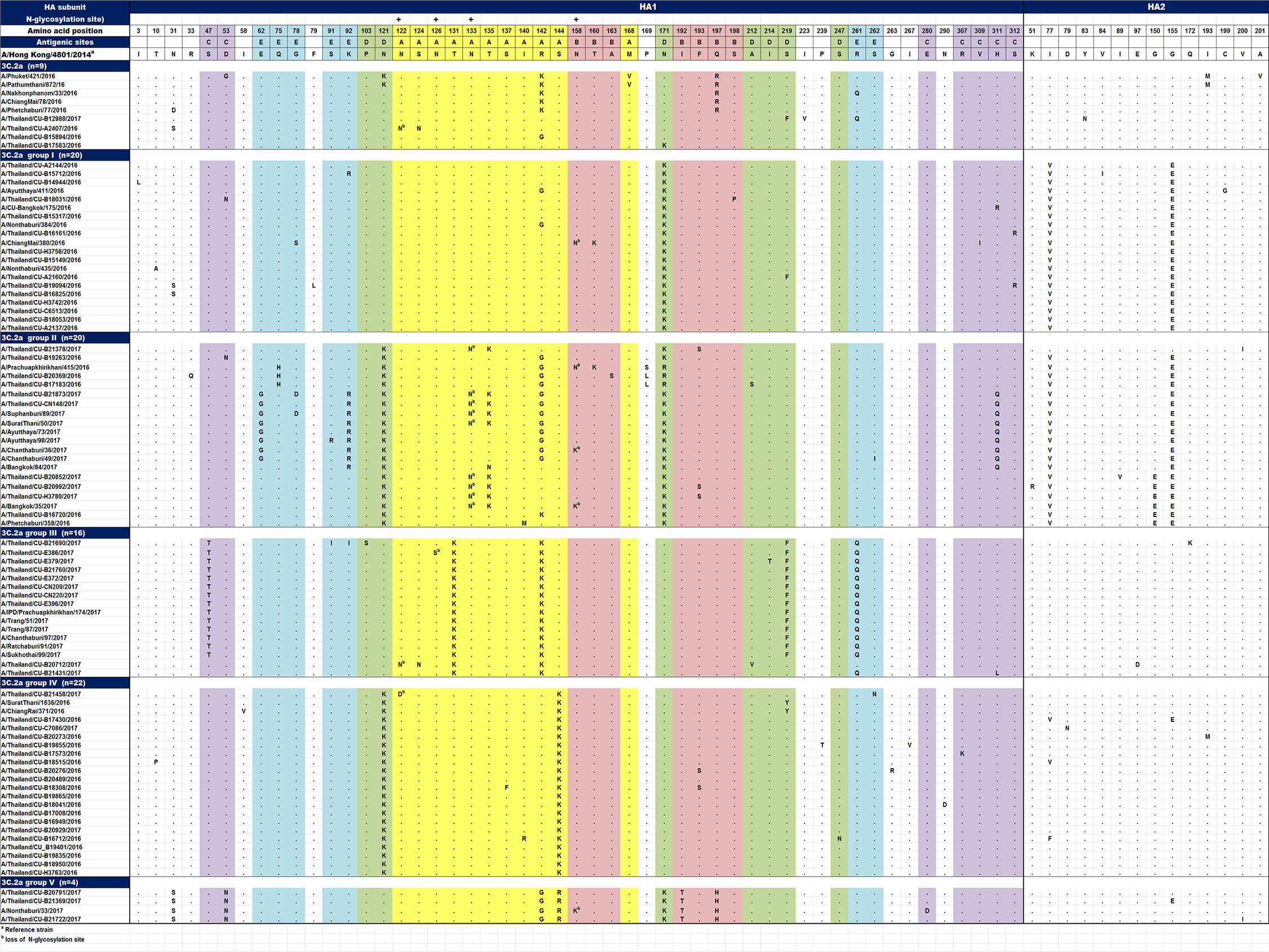

Supplement: S1 Fig — Dominant epitopes (A through E) for each sequence are denoted in different colors (A = yellow, B = pink, C = purple, D = green, E = blue). (TIF) [file pone.0189511.s001.tif]
