# Supplementary material for: Genetic and antigenic divergence in the influenza A(H3N2) virus circulating between 2016 and 2017 in Thailand
Source: PLoS One. 2017 Dec 18;12(12):e0189511. doi: 10.1371/journal.pone.0189511 (PMC5734729; doi:10.1371/journal.pone.0189511)
Supplement: S1 Table — (DOCX) [file pone.0189511.s002.docx]

**S1 Table. Influenza A(H3N2) virus strains sequenced in this study.**

| **Strains** | **Collection-date (DD-MM-YYYY)** | **Location** | **Age(yr)** | **Sex** | **Accession numbers (HA gene)** |
| --- | --- | --- | --- | --- | --- |
| A/Thailand/CU-A2137/2016 | 7-Jan-2016 | Khon Kaen | 87 | Male | MF673231 |
| A/Thailand/CU-A2144/2016 | 7-Jan-2016 | Khon Kaen | 63 | Female | MF673232 |
| A/Thailand/CU-A2160/2016 | 7-Jan-2016 | Khon Kaen | 45 | Female | MF673233 |
| A/Thailand/CU-B14944/2016 | 1-Feb-2016 | Bangkok | 25 | Female | MF673234 |
| A/Thailand/CU-B15149/2016 | 15-Feb-2016 | Bangkok | 12 | Female | MF673235 |
| A/Thailand/CU-B15317/2016 | 23-Feb-2016 | Bangkok | 54 | Female | MF673236 |
| A/Thailand/CU-B15712/2016 | 15-Mar-2016 | Bangkok | 18 | Male | MF673237 |
| A/Thailand/CU-B15894/2016 | 25-Mar-2016 | Bangkok | 70 | Female | MF673238 |
| A/Thailand/CU-B16161/2016 | 7-Apr-2016 | Bangkok | 53 | Female | MF673239 |
| A/Thailand/CU-C6513/2016 | 21-Apr-2016 | Khon Kaen | 7 | Female | MF673240 |
| A/Thailand/CU-A2407/2016 | 18-May-2016 | Khon Kaen | 80 | Male | MF673241 |
| A/Thailand/CU-B16712/2016 | 16-Jun-2016 | Bangkok | 53 | Male | MF673242 |
| A/Thailand/CU-B16720/2016 | 20-Jun-2016 | Bangkok | 16 | Male | MF673243 |
| A/Thailand/CU-B16825/2016 | 5-Jul-2016 | Bangkok | 57 | Female | MF673244 |
| A/Thailand/CU-B16949/2016 | 9-Jul-2016 | Bangkok | 70 | Female | MF673245 |
| A/Thailand/CU-B17008/2016 | 21-Jul-2016 | Bangkok | 35 | Male | MF673246 |
| A/Thailand/CU-B17183/2016 | 25-Jul-2016 | Bangkok | 33 | Female | MF673247 |
| A/Thailand/CU-H3742/2016 | 28-Jul-2016 | Bangkok | 4 | Female | MF673248 |
| A/Thailand/CU-B17430/2016 | 9-Aug-2016 | Bangkok | 50 | Male | MF673249 |
| A/Thailand/CU-B17573/2016 | 10-Aug-2016 | Bangkok | 102 | Female | MF673250 |
| A/Thailand/CU-B17583/2016 | 11-Aug-2016 | Bangkok | 28 | Female | MF673251 |
| A/Thailand/CU-B18031/2016 | 2-Sep-2016 | Bangkok | 38 | Female | MF673252 |
| A/Thailand/CU-B18041/2016 | 3-Sep-2016 | Bangkok | 28 | Female | MF673253 |
| A/Thailand/CU-B18053/2016 | 5-Sep-2016 | Bangkok | 59 | Female | MF673254 |
| A/Thailand/CU-B18308/2016 | 12-Sep-2016 | Bangkok | 13 | Male | MF673255 |
| A/Thailand/CU-B18515/2016 | 19-Sep-2016 | Bangkok | 4 | Female | MF673256 |
| A/Thailand/CU-H3756/2016 | 13-Sep-2016 | Bangkok | 66 | Female | MF673257 |
| A/Thailand/CU-H3763/2016 | 26-Sep-2016 | Bangkok | 13 | Male | MF673258 |
| A/Thailand/CU-B18950/2016 | 4-Oct-2016 | Bangkok | 81 | Female | MF673259 |
| A/Thailand/CU-B19094/2016 | 7-Oct-2016 | Bangkok | 51 | Male | MF673260 |
| A/Thailand/CU-B19263/2016 | 15-Oct-2016 | Bangkok | 4 | Male | MF673261 |
| A/Thailand/CU-B19401/2016 | 19-Oct-2016 | Bangkok | 50 | Female | MF673262 |
| A/Thailand/CU-B19835/2016 | 10-Nov-2016 | Bangkok | 52 | Male | MF673263 |
| A/Thailand/CU-B19855/2016 | 12-Nov-2016 | Bangkok | 1 | Female | MF673264 |
| A/Thailand/CU-B19865/2016 | 15-Nov-2016 | Bangkok | 60 | Female | MF673265 |
| A/Thailand/CU-B20273/2016 | 3-Dec-2016 | Bangkok | 10 | Female | MF673266 |
| A/Thailand/CU-B20276/2016 | 6-Dec-2016 | Bangkok | 10 | Male | MF673267 |
| A/Thailand/CU-B20369/2016 | 9-Dec-2016 | Bangkok | 17 | Female | MF673268 |
| A/Thailand/CU-B20489/2016 | 20-Dec-2016 | Bangkok | 37 | Male | MF673269 |
| A/Thailand/CU-B20712/2017 | 5-Jan-2017 | Bangkok | 19 | Female | MF673270 |
| A/Thailand/CU-B20791/2017 | 14-Jan-2017 | Bangkok | 39 | Male | MF673271 |
| A/Thailand/CU-B20852/2017 | 18-Jan-2017 | Bangkok | 11 | Female | MF673272 |
| A/Thailand/CU-B20929/2017 | 26-Jan-2017 | Bangkok | 64 | Female | MF673273 |
| A/Thailand/CU-B20992/2017 | 4-Feb-2017 | Bangkok | 8 | Male | MF673274 |
|  |  |  |  |  | *(Continued)* |

**S1 Table:** *(Continued)*

| **Strains** | **Collection-date (DD-MM-YYYY)** | **Location** | **Age(yr)** | **Sex** | **Accession numbers (HA gene)** |
| --- | --- | --- | --- | --- | --- |
| A/Thailand/CU-C7086/2017 | 1-Feb-2017 | Khon Kaen | 1 | Female | MF673275 |
| A/Thailand/CU-H3780/2017 | 7-Feb-2017 | Bangkok | 9 | Male | MF673276 |
| A/Thailand/CU-B21369/2017 | 1-Mar-2017 | Bangkok | 31 | Female | MF673277 |
| A/Thailand/CU-B21378/2017 | 4-Mar-2017 | Bangkok | 5 | Female | MF673278 |
| A/Thailand/CU-B21431/2017 | 5-Mar-2017 | Bangkok | 72 | Male | MF673279 |
| A/Thailand/CU-B21458/2017 | 11-Mar-2017 | Bangkok | 1 | Male | MF673280 |
| A/Thailand/CU-B21690/2017 | 6-Apr-2017 | Bangkok | 29 | Male | MF673281 |
| A/Thailand/CU-B21722/2017 | 12-Apr-2017 | Bangkok | 38 | Male | MF673282 |
| A/Thailand/CU-B21760/2017 | 19-Apr-2017 | Bangkok | 54 | Female | MF673283 |
| A/Thailand/CU-CN148/2017 | 26-Apr-2017 | Khon Kaen | 1 | Male | MF673284 |
| A/Thailand/CU-B21873/2017 | 15-May-2017 | Bangkok | 52 | Female | MF673285 |
| A/Thailand/CU-CN209/2017 | 30-May-2017 | Khon Kaen | 27 | Male | MF673286 |
| A/Thailand/CU-CN220/2017 | 30-May-2017 | Khon Kaen | 7 | Female | MF673287 |
| A/Thailand/CU-E372/2017 | 30-May-2017 | Khon Kaen | 69 | Female | MF673288 |
| A/Thailand/CU-E379/2017 | 30-May-2017 | Khon Kaen | 12 | Male | MF673289 |
| A/Thailand/CU-B21988/2017 | 1-Jun-2017 | Bangkok | 5 | Male | MF673290 |
| A/Thailand/CU-E386/2017 | 22-Jun-2017 | Khon Kaen | 29 | Male | MF673291 |
| A/Thailand/CU-E396/2017 | 22-Jun-2017 | Khon Kaen | 34 | Female | MF673292 |
