# Supplementary material for: Genetic and antigenic divergence in the influenza A(H3N2) virus circulating between 2016 and 2017 in Thailand
Source: PLoS One. 2017 Dec 18;12(12):e0189511. doi: 10.1371/journal.pone.0189511 (PMC5734729; doi:10.1371/journal.pone.0189511)
Supplement: S2 Table — (DOCX) [file pone.0189511.s003.docx]

**S2 Table. Accession numbers in GenBank and GISAID of HA influenza A(H3N2) and gene sequences used for phylogenetic tree analysis.**

| **Name** | **Accession Numbers** | | | |  | |  | |
| --- | --- | --- | --- | --- | --- | --- | --- | --- |
| A/H3N2 reference and vaccine strains | EPI545333 | | EPI467994 | | EPI513286 | | EPI630781 | |
|  | EPI577972 | | EPI460558 | | EPI1015612 | | EPI641435 | |
|  | EPI547993 | | EPI814528 | | EPI326139 | | EPI831633 | |
|  | EPI574644 | | EPI769485 | | EPI346607 | | EPI831759 | |
|  | EPI984070 | | EPI426061 | | EPI1026711 | | EPI699750 | |
|  | EPI551882 | | EPI746057 | | EPI550842 | | EPI831876 | |
| A/H3N2-TH strains (2016) | EPI919303 | EPI813825 | | EPI925087 | | EPI836924 | |  |
|  | EPI919249 | | EPI813865 | | EPI925071 | | EPI919402 | |
|  | EPI919405 | | EPI813857 | | EPI825071 | | EPI925111 | |
|  | EPI919411 | | EPI925047 | |  | |  | |
| A/H3N2-TH strains (2017) | EPI1044759 | | EPI1058585 | | EPI1058593 | | EPI1058213 | |
|  | EPI1045321 | | EPI1058173 | | EPI1058221 | | EPI1058165 | |
|  | EPI1058561 | | EPI1044762 | | EPI1058523 | | EPI1044765 | |
|  | EPI1058569 | | EPI1058157 | | EPI1058553 | |  | |
| A/H3N2-Canada strains (2016-17) | EPI953884 | | EPI953827 | | EPI953856 | | EPI1033751 | |
|  | EPI953911 | | EPI953908 | | EPI953698 | |  | |
| A/H3N2-Greece strains (2016-17) | EPI884614 | | EPI901680 | |  | |  | |
| A/H3N2-Denmark strains (2016-17) | EPI879358 | | EPI879380 | | EPI879349 | |  | |
| A/H3N2-Israel strains (2016-17) | EPI942316 | | EPI942315 | |  | |  | |
| A/H3N2-London strains (2016-17) | From [30]. | |  | |  | |  | |
