# Supplementary material for: Genetic and antigenic divergence in the influenza A(H3N2) virus circulating between 2016 and 2017 in Thailand
Source: PLoS One. 2017 Dec 18;12(12):e0189511. doi: 10.1371/journal.pone.0189511 (PMC5734729; doi:10.1371/journal.pone.0189511)
Supplement: S3 Table — (DOCX) [file pone.0189511.s004.docx]

| **S3 Table. Positive selected sites on coding sequences of HA among influenza A(H3N2) circulating in Thailand between January 2016 and June 2017.** | | | | | | | |
| --- | --- | --- | --- | --- | --- | --- | --- |
|  |  |  |  | |  |  |  |
|  | **SLAC** |  | **FEL** | |  | **MEME** |  |
| **Codon** | ***dN*/d*S*** | ***p*-value** | ***dN*/d*S*** | | ***p*-value** | **ω^+^** | ***p*-value** |
| 131 |  |  |  | |  | >100 | **0.001** |
|  |  |  |  | |  |  |  |
| 142 |  |  | 56.21 | | 0.07 |  |  |
| 144 |  |  |  | |  | >100 | **0.036** |
| 171 |  |  | 52 | | 0.06 | >100 | 0.089 |
| 261 |  |  | 46.76 | | 0.07 | >100 | 0.095 |
| 406 |  |  | 41.34 | | 0.08 |  |  |
| 416 |  |  |  | |  | >100 | 0.074 |
| d*N*/d*S* or ω is the ratio of non-synonymous to synonymous substitutions. | | | | | |  |  |
| *p*-value from the SLAC, FEL, and MEME results for positive selection sites. | | | | | |  |  |
| The statistically significant values are shown in bold. | | | |  | |  |  |
