# Supplementary material for: Genetic and antigenic divergence in the influenza A(H3N2) virus circulating between 2016 and 2017 in Thailand
Source: PLoS One. 2017 Dec 18;12(12):e0189511. doi: 10.1371/journal.pone.0189511 (PMC5734729; doi:10.1371/journal.pone.0189511)
Supplement: S4 Table — (DOCX) [file pone.0189511.s005.docx]

**S4 Table. Calculated vaccine efficacy using *P*_epitope_ model and number of mutations in dominant epitope of influenza A(H3N2) circulating in Thailand during January,2016 to June, 2017.**

| **Vaccine strain** | **Year** | **Dominant epitope** | **No. mutation** | **No. of strain** | ***P*_epitope_** | **vaccine efficacy(%)** |
| --- | --- | --- | --- | --- | --- | --- |
| A/Hong Kong/4801/2014 | 2016 (n=53) | A | 1 | 30 | 0.053 | 72.34 |
|  |  |  | 2 | 4 | 0.105 | 44.68 |
|  |  | B | 1 | 2 | 0.048 | 74.97 |
|  |  | C | 1 | 2 | 0.037 | 80.54 |
|  |  | D | 1 | 13 | 0.024 | 87.18 |
|  |  |  | 3 | 1 | 0.073 | 61.55 |
|  |  | E | 1 | 1 | 0.046 | 76.11 |
|  | **Mean** |  |  |  | **0.049** | **74.17** |
| A/Hong Kong/4801/2014 | 2017 (n=38) | A | 1 | 7 | 0.053 | 72.34 |
|  |  |  | 2 | 21 | 0.105 | 44.68 |
|  |  |  | 3 | 2 | 0.158 | 17.02 |
|  |  | B | 2 | 1 | 0.095 | 49.94 |
|  |  |  | 3 | 1 | 0.143 | 24.92 |
|  |  | E | 1 | 1 | 0.046 | 76.11 |
|  |  |  | 2 | 2 | 0.091 | 52.22 |
|  |  |  | 3 | 3 | 0.136 | 28.34 |
|  | **Mean** |  |  |  | **0.099** | **47.87** |
